# High-frequency oscillatory ventilation with sigh breath increases pneumothorax in neonates born at 22–25 gestational weeks

**DOI:** 10.1186/s12887-025-06142-1

**Published:** 2025-10-22

**Authors:** Tomonori Kurimoto, Takuya Tokuhisa, Asataro Yara, Masaya Kibe, Hiroshi Ohashi, Eiji Hirakawa, Takatsugu Maeda, Masato Kamitomo

**Affiliations:** 1 Department of Neonatology, Perinatal Medical Center, Kagoshima City Hospital, Kagoshima, Japan; 2 Department of Obstetrics and Gynecology, Perinatal Medical Center, Kagoshima City Hospital, Kagoshima, Japan

**Keywords:** 22–25 weeks of gestation, Extremely preterm neonates, High-frequency oscillatory ventilation, Sigh breaths, Tension pneumothorax

## Abstract

**Background:**

High-frequency oscillatory ventilation (HFOV) is widely used in neonates with respiratory distress syndrome (RDS) to optimize lung recruitment while minimizing ventilator-induced lung injury. Sigh breaths have been incorporated into HFOV to improve alveolar recruitment in cases of atelectasis. However, the safety of this approach, particularly in extremely preterm neonates, remains unclear. This study aimed to evaluate whether the use of sigh breaths during HFOV increased the risk of developing tension pneumothorax within the first 96 h after birth in neonates born at 22–25 weeks of gestation with RDS.

**Methods:**

This retrospective cohort study included neonates born at 22–25 weeks of gestation between 2014 and 2023 who required rescue HFOV within 4 h of birth due to respiratory acidosis. Among 66 eligible neonates, 2 were excluded due to congenital anomalies, leaving 64 included for analysis. The study population was categorized into three groups: (1) neonates with atelectasis who received sigh breaths (*n* = 16), (2) neonates with atelectasis who did not receive sigh breaths (*n* = 3), and (3) neonates without atelectasis who did not receive sigh breaths (*n* = 45). The primary outcome was the incidence of tension pneumothorax within 12–96 h post-birth.

**Results:**

Tension pneumothorax occurred in 10 neonates (15.6%). Bivariable analysis revealed a significant association between the use of sigh breaths and tension pneumothorax (*p* = 0.007). Firth’s penalized logistic regression demonstrated that neonates with atelectasis who received sigh breaths had a significantly higher risk of developing tension pneumothorax (odds ratio = 5.5, 95% confidence interval: 1.2–23.9, *p* = 0.02) compared to those without atelectasis who did not receive sigh breaths. In contrast, no significant difference was observed between neonates with and without atelectasis who did not receive sigh breaths.

**Conclusions:**

While sigh breaths during HFOV may facilitate alveolar recruitment in preterm neonates with atelectasis, their use appears to significantly increase the risk of tension pneumothorax. Given the vulnerability of extremely preterm lungs, caution is required when implementing sigh breaths in this population. Further prospective studies are needed to refine ventilation strategies and minimize complications in extremely preterm neonates.

**Supplementary Information:**

The online version contains supplementary material available at 10.1186/s12887-025-06142-1.

## Background

High-frequency oscillatory ventilation (HFOV) is widely utilized as a mechanical ventilation strategy to maintain sufficient alveolar ventilation with low tidal volumes, thereby minimizing ventilator-associated lung injuries [1]. HFOV can be employed as both an elective and a rescue therapy and is particularly advantageous for protecting the fragile lungs of preterm neonates [1]. Elective HFOV is proactively applied to stabilize respiratory function and prevent deterioration in neonates at risk of severe respiratory distress syndrome (RDS), contributing not only to immediate respiratory improvements but also to long-term respiratory health [2]. In contrast, rescue HFOV is used when conventional mechanical ventilation (CMV) fails to achieve adequate gas exchange [3].

While HFOV has been shown to maintain lung recruitment and reduce lung injury, concerns remain regarding its association with pulmonary air leaks. A 2015 meta-analysis of 19 trials involving 4,096 preterm infants reported a higher incidence of pulmonary air leaks in the HFOV group compared to the CMV group [2]. Despite this, mortality rates and incidences of severe intraventricular hemorrhage (IVH) or periventricular leukomalacia were comparable between the two groups [3].

To further optimize lung recruitment and ventilation efficiency during HFOV, sigh breaths have been introduced as an adjunctive maneuver. Sigh breaths are intermittent high-pressure inflations designed to recruit collapsed alveoli, improve end-expiratory lung volume (EELV), and enhance oxygenation [4]. A randomized crossover study demonstrated that intermittent sigh breaths during HFOV significantly improved oxygenation and lung compliance in preterm neonates [5]. Electrical impedance tomography (EIT) data showed increased ventilation in the posterior lung regions, correlating with significant improvements in oxygen saturation (*p* < 0.01) [5]. These findings underscore the physiological benefits of incorporating sigh breaths into HFOV management.

However, the safety profile of sigh breaths during HFOV remains controversial, particularly regarding their impact on pulmonary air leaks such as tension pneumothorax [6]. While effective in recruiting alveoli, sigh breaths can transiently increase airway pressures, potentially predisposing immature, fragile lungs to overdistension, alveolar rupture, and air leaks [6]. Currently, no systematic studies have evaluated the relationship between HFOV with sigh breaths and the risk of tension pneumothorax within the first 96 h of life in extremely preterm neonates born at 22–25 weeks of gestation after RDS treatment.

This study aimed to investigate whether the incorporation of sigh breaths during rescue HFOV increased the risk of tension pneumothorax within 96 h of birth in extremely preterm neonates treated for RDS. By analyzing clinical outcomes and exploring the physiological effects of sigh breaths, we sought to determine whether this intervention provided net benefits or posed risks in this vulnerable population.

## Methods

### Study design and population

This study was a retrospective cohort investigation conducted at a single tertiary medical center in Kagoshima Prefecture, Japan. The medical center serves as a referral hub for maternal and neonatal cases from primary and secondary perinatal facilities in the region. Expectant mothers expected to deliver between 22 weeks + 0 days and 25 weeks + 6 days of gestation were transferred to our tertiary medical center for admission and management whenever possible. In situations where timely maternal transfer was impractical, neonates were transported to the hospital within 2 h of birth. All neonates received care from a specialized medical team following an institutional treatment protocol [7].

The study cohort comprised neonates born between January 2014 and March 2023 at gestational ages of 22 weeks + 0 days to 25 weeks + 6 days who were admitted to the neonatal intensive care unit (NICU) within 2 h of birth and with HFOV initiated within 4 h of delivery. Rescue HFOV was employed for neonates who developed persistent respiratory acidosis, defined as an arterial blood gas pH < 7.2 and PaCO2 > 65 mmHg, despite receiving maximal respiratory support with synchronized intermittent mandatory ventilation (SIMV).

Neonates with chromosomal abnormalities, such as trisomy 21, or major congenital anomalies, including aortopulmonary window or ventricular septal defects, were excluded. Using the FileMaker Pro database management system (Claris International Inc., Santa Clara, CA, USA), 66 consecutive cases were identified during the study period. After applying exclusion criteria, 64 neonates (24.8%) were eligible for inclusion.

### Study outcomes

Among the 64 neonates who underwent a transition to rescue HFOV within 4 h after birth, we conducted a comparative analysis between a group of 10 neonates who developed tension pneumothorax within 12–96 h of life and the rest who did not. Subsequently, we aimed to identify the risk factors for tension pneumothorax using multivariate analyses.

### Definitions and treatment

#### Rescue HFOV

In all cases, upon admission, ventilation was initiated in the SIMV (VN500 ventilator; Dräger, Lübeck, Germany) mode, and fractions of inspired oxygen (FiO_2_) were maintained at postductal arterial oxygen saturation of 90–95% [8, 9]. All cases were treated with surfactants for RDS. Surfactant (poractant alfa, 120 mg/kg) was administered via endotracheal tube during synchronized intermittent mandatory ventilation (SIMV) when the FiO₂ exceeded 0.40 and respiratory acidosis persisted. In most cases, administration occurred within 1–2 h after birth. Despite SIMV mode settings at peak inspiratory pressure of 20–21 cmH_2_O, positive end-expiratory pressure of 6 cmH_2_O, and respiratory rate of 60–65/min, respiratory acidosis with arterial blood gas findings of pH < 7.2 and PaCO_2_ > 65 mmHg was maintained [10]. In cases where the response to SIMV was deemed insufficient, as indicated by an inability to decrease PCO_2_ by > 10% and/or FiO_2_ by > 20% within 1 h of initiating SIMV, we opted to transition to HFOV (i.e., rescue HFOV) [11]. The mean airway pressure (MAP) was initiated at 2–3 cmH_2_O above that on SIMV, but had to be sufficient to inflate the lungs (i.e., with complete white-out and stiff lungs, a markedly higher MAP was required). A frequency range of 12–15 Hz and an inspiration: expiration ratio of 1:1 was used. If adequate PaCO_2_ was not achieved with the maximum amplitude, the frequency decreased to 10 Hz [1]. The volume guarantee (VG) mode was managed with a target range of 1.5–2.0 mL/kg [12].

In cases where radiographic examination revealed atelectasis, transitioning to HFOV was accompanied by the administration of sigh breaths to aid in lung recruitment and reduce atelectasis. This intervention was introduced into clinical practive at our institution in January 2015 and implemented according to a standardized institutional protocol [13]. Sigh breaths were delivered at a frequency of 1–5 breaths/min, with the inspiratory pressure and duration set at 30 cmH_2_O and 1.0 s, respectively, based on prior evidence [5].

The administration of sigh breaths was discontinued during weaning from respiratory ventilation support and the transition from HFOV to SIMV. Termination of sigh breaths occurred upon the identification of pulmonary interstitial emphysema or pneumothorax findings on radiography.

To assess the potential influence of temporal changes in clinical practice, such as ventilation strategies and surfactant administration protocols, we conducted a univariable analysis comparing neonates born in 2014 (*n* = 7) and those born from 2015 to 2023 (*n* = 57). Clinical characteristics and the incidence of tension pneumothorax were compared between these two cohorts (Supplementary Table 1).

#### Pulmonary hypertension

Echocardiographic evidence of pulmonary hypertension is defined as an estimated peak systolic pulmonary artery pressure of ≥ 35 mmHg, which exceeds two-thirds of the systemic systolic pressure. This was indicated by the presence of a tricuspid regurgitation jet, right-to-left patent ductus arteriosus shunt, or right-to-left artery-level shunt [14].

#### Tension pneumothorax

Tension pneumothorax development between 12 and 96 h after birth was diagnosed if radiographic examination revealed any displacement of the affected lung from the chest wall based on the appearance of a radiologically luminescent air band. Additional diagnostic criteria included diaphragmatic depression, contralateral mediastinal shift, and transillumination of the posterior axillary line in suspected cases of lateral luminescent tension pneumothorax. Radiographs were reviewed by two board-certified neonatologists with reference to standardized diagnostic criteria. In cases of interpretive disagreement, final diagnosis was determined by consensus discussion. After diagnosis, treatment was performed with fine-needle aspiration and insertion of a chest tube.

### Statistical analyses and sample size calculation

Statistical analyses were conducted to evaluate the association between the use of sigh breaths and the development of tension pneumothorax in neonates born at 22–25 weeks of gestation. A total of 64 neonates were divided into three groups: Group A (*n* = 16, atelectasis with sigh breaths), Group B (*n* = 3, atelectasis without sigh breaths), and Group C (n = 45, no atelectasis or sigh breaths, control).

A post hoc power analysis was performed to confirm the adequacy of the sample size. Based on an observed event rate of 15.6% in Group C and an odds ratio of 5.5 for Group A vs. Group C, the effect size (Cohen’s w) was calculated as 0.802, indicating a large effect size. With a total sample size of 64, the statistical power was approximately 100% (α = 0.05). These calculations were performed using Python (version 3.13, 64-bit) and the statsmodels package.

Initial comparisons between the tension pneumothorax group (*n* = 10) and the non-tension pneumothorax group (*n* = 54) were performed using Fisher’s exact test. Bivariate analysis identified a significant difference in the use of sigh breaths between these groups. Further analysis compared the three groups (A, B, and C) to investigate the impact of sigh breaths on tension pneumothorax risk. For continuous variables, the Kruskal–Wallis test was used due to non-normal distributions (assessed by the Shapiro–Wilk test), while categorical data were analyzed with the Chi-square test. Bonferroni correction was applied to adjust for multiple comparisons, and both corrected and uncorrected p-values were reported.

To address the small sample size and low event numbers, Firth’s penalized logistic regression was employed. The dependent variable was the presence of tension pneumothorax (0: absent, 1: present), with independent variables including Group (A, B, C) and cesarean section (yes/no), using Group C as the reference. Odds ratios (ORs) and 95% confidence intervals (CIs) were calculated. Sensitivity analyses were conducted using two additional models: Model 1 (Group and gestational week) and Model 2 (Group and birth weight). Statistical analyses were performed using R (version 4.3.1) with the brglm2 package. Given the small sample size of Group B (n = 3), which limits statistical power, we also conducted an exploratory analysis combining Groups A and B (atelectasis with or without sigh breaths) versus Group C (no atelectasis or sigh breaths). (Supplmentary Table 2). 

## Results

Between January 2014 and March 2023, 66 out of 260 neonates born at 22–25 weeks of gestation were admitted to the NICU who had developed persistent respiratory acidosis (pH < 7.2 and PaCO2 > 65 mmHg) despite receiving maximal respiratory support with SIMV. After excluding two neonates with congenital anomalies, 64 neonates were included in the study. Their ventilation mode was switched to HFOV within 4 h after birth. Analyses were conducted on 10 neonates in the tension pneumothorax group and 54 neonates in the non-tension pneumothorax group.

The analyses revealed significant differences in the use of sigh breaths, but no notable differences in perinatal characteristics, clinical variables, or morbidity (Table 1). Oxygenation Index (OI) at the time of HFOV initiation did not differ significantly between groups (median: 8.1 [6.5–11.3] in pneumothorax group vs. 8.1 [6.5–9.7] in the non-pneumothorax group, *p* = 0.92). SpO₂/FiO₂ ratio was not consistently available for all cases and therefore was not included in the main analysis. In the multiple comparisons of the groups (A, B, C), tension pneumothorax demonstrated a significant difference with a p-value of 0.007 (Bonferroni corrected: 0.02).

**Table 1:** Comparison of perinatal and clinical characteristics between neonates with and without tension pneumothorax

Variable	Tension Pneumothorax (*n* = 10)	No Tension Pneumothorax (*n* = 54)	*p*-value
GA (weeks)	24 [23–24]	23 [23–24]	0.48
Sex (male)	7 (70.0%)	31 (57.4%)	0.51
BW (g)	632 [606–702]	568 [457–767]	0.2
FGR	1 (10.0%)	13 (24.1%)	0.44
Outborn	0 (0.0%)	2 (3.7%)	1
C/S	8 (80.0%)	50 (92.6%)	0.23
Antenatal steroids	6 (60.0%)	30 (55.6%)	1
MgSO4	2 (20.0%)	16 (29.6%)	0.71
PROM	2 (20.0%)	13 (24.1%)	1
CAM stage 2–3	6 (60.0%)	31 (57.4%)	1
Funisitis stage 2–3	3 (30.0%)	21 (38.9%)	0.73
Oligohydramnios	3 (30.0%)	9 (16.7%)	0.38
Fetal bradycardia	0 (0.0%)	4 (7.4%)	1
APS at 1 min	2 [1–3]	2 [1–3]	0.34
APS at 5 min	5 [2–6]	6 [3–7]	0.3
UA pH	7.26 [7.13–7.38]	7.30 [7.25–7.37]	0.44
Death	6 (60.0%)	16 (29.6%)	0.08
Severe IVH	2 (20.0%)	7 (13.0%)	0.62
VG mode	6 (60.0%)	34 (63.0%)	1
Sigh breaths	7 (70.0%)	9 (16.7%)	0.001
Fentanyl	2 (20.0%)	26 (48.1%)	0.17
PPHN	1 (10.0%)	5 (9.3%)	1
Re-admission STA	3 (30.0%)	16 (29.6%)	1
Cardiac massage	2 (20.0%)	1 (1.9%)	0.06
IND/IBU	4 (40.0%)	36 (66.7%)	0.11
PDA surgery	0	1(1.9%)	0.66
FiO2 (admission STA)	0.5 (0.4- 0.75)	0.5 (0.4- 0.6)	0.72
OI (admission STA)	8.1 (6.5- 11.3)	8.1 (6.5- 9.7)	0.92
HFO mode (admission STA)	3 (30.0%)	13 (24.1%)	0.71

Values are presented as median [IQR] or n (%). Statistical significance was set at *p* < 0.05

*IQR* interquartile range, *GA* gestational age, *BW* birth weight, *FGR* fetal growth restriction (≤ 10th percentile), *C/S* cesarean section, *MgSO4* Magnesium sulfate, *PROM* premature rupture of membranes, *CAM* chorioamnionitis, *APS* Apgar score, *UA pH* umbilical artery pH, *IVH* intraventricular hemorrhage, *VG* volume guarantee, *PPHN* persistent pulmonary hypertension of the newborn, *STA* surfactant, *IND/IBU* indomethacin/ibuprofen, *PDA* patent ductus arteriosus, *FiO2* fraction of inspired oxygen, *OI* oxygenation index, *HFO* high-frequency oscillation

Because Group B comprised only three neonates, statistical comparisons involving this group were limited. When Groups A and B were combined and compared with Group C (A + B vs. C), no significant association with tension pneumothorax was observed (*p* = 0.13). In contrast, the comparison between Group A and Group C showed a statistically significant difference (*p* = 0.007, Bonferroni corrected *p* = 0.022), suggesting that the increased risk was primarily associated with the use of sigh breaths rather than atelectasis alone.

**Fig. 1:**
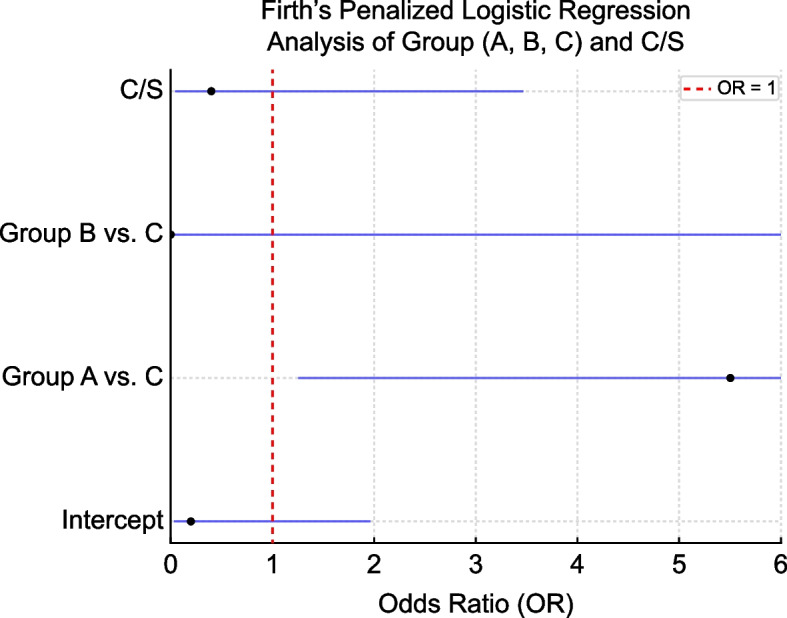
Forest plot of odds ratios (ORs) from Firth’s Penalized Logistic Regression. The risk of tension pneumothorax is significantly higher in Group A than in Group C [OR: 5.5, 95% CI: 1.2–23.9, *p* = 0.02]. No significant associations are observed for Group B or cesarean section (C/S). Error bars indicate 95% CI. CI, confidence interval

For cesarean section (C/S), the p-value was 0.05 (Bonferroni corrected: 0.14), which was not statistically significant. No significant differences were observed in other comparisons (Table 2). Using Group C as the control in Firth’s Penalized Logistic Regression, Group A showed a significant association with an OR of 5.5 (95% CI: 1.2–23.9, *p* = 0.02), indicating a higher event risk compared to Group C. In contrast, Group B did not demonstrate any significant association (OR: 1.7*10^–8^, 95% CI: 0–inf, *p* = 0.99). Similarly, C/S showed no significant association with the event risk (OR: 0.42, 95% CI: 0.05–3.5, *p* = 0.42) (Table 3 and Fig. 1). Sensitivity analyses were performed to assess the robustness of the findings. In Model 1, which included the group (“Group”) and gestational week (“Week”) as covariates, the odds ratio for Week was 1.0. However, the p-value of 0.99 indicated this variable was not statistically significant and unlikely to serve as a meaningful predictor. In Model 2, which included the group (“Group”) and birth weight (BW), the odds ratio for BW was 1.7, suggesting a potential trend toward higher event risk with increasing birth weight. Nonetheless, this finding was not statistically significant (*p* = 0.24) (Table 4).

**Table 2:** Multiple Comparisons of the Groups (A, B, C)

	**Original *p*-value**	**Bonferroni Corrected**
Tension pneumothorax	0.007	0.022
C/S	0.046	0.138

Group B (*n* = 3) was underpowered for statistical comparison. Therefore, the interpretation of comparisons involving Group B should be approached with caution

*C/S* cesarean section

Statistical significance was set at *p* < 0.05. Convergence issues were noted in some models, as indicated by CIs extending to infinity

**Table 3:** Firth’s Penalized Logistic Regression Analysis of Group (A, B, C) and C/S

Variable	OR	CI (95%)	*p*-value
Intercept	0.2	0.03–2.0	0.18
Group A vs. C	5.5	1.3–23.9	0.02
Group B vs. C	1.7 × 10⁻⁸	0-∞	1.00
C/S	0.4	0.05–3.5	0.42

*OR* odds ratio, *CI* confidence interval, *C/S* cesarean section

**Table 4:** Sensitivity Analysis Using Logistic Regression Models

Model	Variable	OR	CI (95%)	*p*-value
Model 1: Group + Gestational weeks	Intercept	0.6	0.21–1.7	0.34
	Gestational weeks	1.0	0.46–2.2	0.99
Model 2: Group + Birth weight	Intercept	0.4	0.14–1.4	0.17
	Birth weight	1.7	0.71–3.9	0.24

*OR* odds ratio, *CI* confidence interval

A univariable analysis comparing neonates born in 2014 (*n* = 7) with those born in 2015–2023 (*n* = 57) revealed no significant differences in gestational age, birth weight, or incidence of tension pneumothorax (2014: 2/7 [28.6%] vs. 2015–2023: 8/57 [14.0%], *p* = 0.29). However, as sigh breaths were not used in 2014, their potential protective or adverse effects could not be assessed in that subgroup. This temporal comparison suggests that the observed association between sigh breaths and pneumothorax is unlikely to be explained solely by changes in clinical practice over time.

## Discussion

This study demonstrated that the addition of sigh breaths to HFOV in extremely preterm infants with RDS who were undergoing treatment increased the risk of developing tension pneumothorax within the first 96 h after birth. Previous meta-analyses also reported a significant increase in the risk of air leaks, including pulmonary interstitial emphysema and overt pneumothorax, in infants treated with HFOV [1].

Sigh breaths play a crucial role in re-expanding collapsed alveoli, optimizing ventilation-perfusion ratios, and enhancing gas exchange efficiency. Atelectasis (collapsed lung) is characterized by decreased compliance and reduced functional residual capacity, both of which can be improved by the application of sigh breaths. The large tidal volumes associated with sigh breaths reopen collapsed alveoli, potentially preventing or resolving atelectasis. This re-expansion of alveolar units improve oxygenation and ensured more effective oxygen delivery to the bloodstream [2–4]. Recent research by Hough et al. demonstrated that intermittent sigh breaths during HFOV increased EELV in the posterior and left lung regions and improved oxygen saturation in preterm infants [5]. Their study, however, focused on preterm infants with a median gestational age of 25 weeks (range: 23.86–30.86 weeks) and a median postnatal age of 25 days (range: 3–49 days). In contrast, our study focused on extremely low birth weight infants born at 22–25 weeks’ gestation with RDS and persistent respiratory acidosis and atelectasis within the first 12–96 h of life. Given these differences in study populations and clinical conditions, caution is required when directly comparing the two studies. Recent guidelines advocate for the use of elective HFOV with volume guarantee (HFOV-VG), combined with individualized lung recruitment maneuvers using incremental and decremental MAP adjustment, to optimize lung volume and reduce ventilator-induced lung injury [15]. These open-lung strategies aim to achieve adequate alveolar inflation at the lowest possible pressure, minimizing barotrauma while improving oxygenation. In contrast, our application of sigh breaths in the acute phase of respiratory failure represents a non-standardized, high-pressure approach that may deviate from these lung-protective strategies. The additional volume and transient pressure surges associated with sigh breaths may predispose the structurally immature and surfactant-deficient lungs of extremely preterm infants to alveolar rupture, potentially explaining the increased incidence of tension pneumothorax observed in our cohort.

The findings of our study suggest that the use of sigh breaths during HFOV may increase the risk of tension pneumothorax due to the combined mechanical stress of HFOV and the additional pressure from sigh breaths. Animal models showed that improper synchronization and excessive tidal volumes could cause airway overdistension, leading to pulmonary interstitial emphysema and alveolar rupture [6]. In extremely preterm infants with RDS, surfactant therapy administered shortly after birth can result in uneven alveolar recruitment, leaving the lungs vulnerable to excessive pressure and ventilation volumes. Without proper synchronization or lung recruitment strategies, the application of sigh breaths during HFOV may exacerbate alveolar injury and increase the risk of tension pneumothorax.

This study has several limitations. First, the findings may not be generalizable to neonates born at > 25 weeks of gestation or those treated after 96 h of life. Second, as an observational study without randomization or blinding, there is a potential for information and recall biases. However, the inclusion of all eligible neonates born at 22–25 weeks of gestation in the study region reduces the risk of selection bias. To enhance the reliability of our findings, we conducted a post hoc power analysis, which indicated a large effect size (Cohen’s w = 0.802) and nearly 100% statistical power (α = 0.05). Sensitivity analyses using multiple models confirmed the consistency of the results. Additionally, we employed Firth’s penalized logistic regression to address potential biases related to small sample sizes and low event rates, ensuring robust and reliable estimates.

These methodological approaches strengthen the validity and reliability of the study’s conclusions. Chest radiographs were reviewed by two board-certified neonatologists using predefined diagnostic criteria, and blinding to clinical information was not feasible due to the integrated nature of care. This may have introduced potential interpretation bias, which should be acknowledged as a limitation. As this was an observational study, a causal relationship between sigh breaths and pneumothorax cannot be definitively established. It is possible that sigh breaths were preferentially applied in more severe cases of atelectasis or respiratory failure, which may themselves carry a higher intrinsic risk of air leak (i.e., indication bias). To address the potential for temporal bias, we compared neonates treated in 2014—before the implementation of sigh breaths—with those treated from 2015 onward. Although the use of sigh breaths was limited to the latter cohort, baseline characteristics and pneumothorax rates did not differ significantly. These findings support the conclusion that the association between sigh breaths and tension pneumothorax is not merely an artifact of evolving clinical practice over time.

The very small sample size of Group B (atelectasis without sigh breaths) limited our ability to draw meaningful statistical inferences for this subgroup. Although we explored a combined analysis of Groups A and B versus Group C, this comparison did not show a statistically significant difference. Our findings suggest that the increased risk of pneumothorax is more likely related to the use of sigh breaths rather than the presence of atelectasis alone. Therefore, we emphasized the more robust and statistically significant comparison between Group A (atelectasis with sigh breaths) and Group C (no atelectasis or sigh breaths). Nonetheless, the limitations due to small sample size and potential confounding must be acknowledged.

In conclusion, this study suggests that the addition of sigh breaths to HFOV in neonates born at 22–25 weeks’ gestation who received surfactant therapy for RDS may increase the risk of developing tension pneumothorax within the first 12–96 h after birth. Prospective randomized controlled trials comparing HFOV with and without sigh breaths are needed to establish causality. Further research is also warranted to identify the optimal ventilator settings to minimize the incidence of pneumothorax and to explore monitoring strategies for the early detection of pneumothorax.

## Supplementary Information

Supplementary Material 1.

## Data Availability

The datasets generated and/or analyzed during the current study are available from the corresponding author on reasonable request.
