# Supplementary material for: High-frequency oscillatory ventilation with sigh breath increases pneumothorax in neonates born at 22–25 gestational weeks
Source: BMC Pediatr. 2025 Oct 22;25:850. doi: 10.1186/s12887-025-06142-1 (PMC12542469; doi:10.1186/s12887-025-06142-1)
Supplement: Supplementary file 1 — Supplementary Material 1. [file 12887_2025_6142_MOESM1_ESM.docx]

**Supplementary Table 1**. Comparison of perinatal and clinical characteristics between neonates born in 2014 and 2015-2023

| **Variable** | **2014 (n=7)** | **2015-2023 (n=57)** | **p-value** |
| --- | --- | --- | --- |
| **GA (weeks)** | 24 [23–24] | 24 [23–24] | 0.39 |
| **Sex (male)** | 3 (42.9%) | 35 (61.4%) | 0.35 |
| **BW (g)** | 618 [591–662] | 568 [478–647] | 0.35 |
| **FGR** | 2(28.6%) | 11(19.2%) | 0.57 |
| **Antenatal steroids** | 5 (71.4%) | 31 (54.4%) | 0.39 |
| **PROM** | 3 (42.9%) | 11 (19.3%) | 0.16 |
| **CAM stage 2–3** | 5 (71.4%) | 32 (56.1%) | 0.44 |
| **Funisitis stage 2–3** | 3 (42.9%) | 21 (36.8%) | 0.76 |
| **Oligohydramnios** | 2 (28.6%) | 10 (17.5%) | 0.48 |
| **APS at 1 min** | 2 [1–2] | 2 [1–3] | 0.52 |
| **APS at 5 min** | 6 [5–6] | 6 [3–7] | 0.87 |
| **UA pH** | 7.202 [7.184–7.333] | 7.299 [7.244–7.363] | 0.07 |
| **Death** | 2 (28.6%) | 20 (35.1%) | 0.73 |
| **VG mode** | 3 (42.9%) | 37 (64.9%) | 0.69 |
| **Sigh breaths** | 0 | 16 (28.1%) | 0.11 |
| **Fentanyl** | 2 (28.6%) | 26 (45.6%) | 0.39 |
| **PPHN** | 1 (14.3%) | 5 (8.8%) | 0.64 |
| **Tension Pneumothorax** | 1(14.3%) | 9(15.8%) | 0.92 |
| **Re-admission STA** | 3 (42.9%) | 17 (29.8%) | 0.48 |
| **Cardiac massage** | 1 (14.3%) | 2 (3.5%) | 0.20 |
| **IND/IBU** | 5 (71.4%) | 35 (61.4%) | 0.61 |
| **PDA surgery** | 0 | 1(1.8%) | 0.72 |
| **FiO2 (admission STA)** | 0.5 (0.4- 0.7) | 0.5 (0.4- 0.6) | 0.70 |
| **OI (admission STA)** | 8.3 (6.5- 11.0) | 8.1 (6.5- 10.0) | 0.17 |
| **HFO mode**  **(admission STA)** | 2 (28.6%) | 14 (24.6%) | 0.81 |

Values are presented as median [IQR] or n (%). Statistical significance was set at p < 0.05.

**Abbreviations:** IQR, interquartile range; GA, gestational age; BW, birth weight; FGR, fetal growth restriction (≤10th percentile); PROM, premature rupture of membranes; CAM, chorioamnionitis; APS, Apgar score; UA pH, umbilical artery pH; VG, volume guarantee; PPHN, persistent pulmonary hypertension of the newborn; STA, surfactant; IND/IBU, indomethacin/ibuprofen; PDA, patent ductus arteriosus; FiO2, fraction of inspired oxygen; OI, oxygenation index; HFO, high-frequency oscillation.

.

**Supplementary Table 2**. Comparison of perinatal and clinical characteristics between neonates with and without atelectasis.

| **Variable** | **Atelectasis (n=19)** | **No Atelectasis (n=45)** | **p-value** |
| --- | --- | --- | --- |
| **GA (weeks)** | 24 [23–24] | 24 [23–24] | 0.59 |
| **Sex (male)** | 9 (47.4%) | 29 (64.4%) | 0.20 |
| **BW (g)** | 568 [531–662] | 580 [510–647] | 0.30 |
| **FGR** | 4 (21.1%) | 10 (28.9%) | 0.92 |
| **Antenatal steroids** | 10 (52.6%) | 26 (57.8%) | 0.71 |
| **PROM** | 7 (36.8%) | 8 (17.8%) | 0.10 |
| **CAM stage 2–3** | 11 (57.9%) | 26 (57.8%) | 0.99 |
| **Funisitis stage 2–3** | 8 (42.1%) | 16 (35.6%) | 0.62 |
| **Oligohydramnios** | 4 (21.1%) | 8 (17.8%) | 0.76 |
| **APS at 1 min** | 2 [1–3] | 2 [1–3] | 0.94 |
| **APS at 5 min** | 6 [4–7] | 5 [3–6] | 0.65 |
| **UA pH** | 7.33 [7.23–7.37] | 7.3 [7.25–7.36] | 0.69 |
| **Death** | 5 (26.3%) | 17 (37.8%) | 0.38 |
| **VG mode** | 10 (52.6%) | 30 (66.7%) | 0.29 |
| **Fentanyl** | 7 (36.8%) | 24 (53.3%) | 0.23 |
| **PPHN** | 1 (5.3%) | 5 (11.1%) | 0.46 |
| **Tension Pneumothorax** | 5 (26.3%) | 5 (11.1%) | 0.13 |
| **Re-admission STA** | 5 (26.3%) | 14 (31.1%) | 0.70 |
| **Cardiac massage** | 1 (5.3%) | 2 (4.4%) | 0.89 |
| **IND/IBU** | 11 (57.9%) | 29 (64.4%) | 0.62 |
| **PDA surgery** | 1 (5.3%) | 0 | 0.12 |
| **FiO2 (admission STA)** | 0.5 (0.4- 0.7) | 0.5 (0.4- 0.6) | 0.85 |
| **OI (admission STA)** | 8.0 (6.6- 10.0) | 8.1 (6.7- 10.5) | 0.26 |
| **HFO mode**  **(admission STA)** | 6 (31.6%) | 10 (22.2%) | 0.43 |

Values are presented as median [IQR] or n (%). Statistical significance was set at p < 0.05.

**Abbreviations:** IQR, interquartile range; GA, gestational age; BW, birth weight; FGR, fetal growth restriction (≤10th percentile); C/S, cesarean section; MgSO4, Magnesium sulfate; PROM, premature rupture of membranes; CAM, chorioamnionitis; APS, Apgar score; UA pH, umbilical artery pH; IVH, intraventricular hemorrhage; VG, volume guarantee; PPHN, persistent pulmonary hypertension of the newborn; STA, surfactant; IND/IBU, indomethacin/ibuprofen; PDA, patent ductus arteriosus; FiO2, fraction of inspired oxygen; OI, oxygenation index; HFO, high-frequency oscillation.

.
